# Experimental Research on Machining Localization and Surface Quality in Micro Electrochemical Milling of Nickel-Based Superalloy

**DOI:** 10.3390/mi9080402

**Published:** 2018-08-14

**Authors:** Yong Liu, Yong Jiang, Chunsheng Guo, Shihui Deng, Huanghai Kong

**Affiliations:** 1 Associated Engineering Research Center of Mechanics and Mechatronic Equipment, Shandong University, Weihai 264209, China; 201736323@mail.sdu.edu.cn (Y.J.); guo@sdu.edu.cn (C.G.); 201614777@mail.sdu.edu.cn (S.D.); 201614780@mail.sdu.edu.cn (H.K.); 2 Suzhou Institute of Shandong University, Room522, Building H of National University Science and Technology Park of Nanotechnology, No.388 Ruoshui Road, Suzhou Industrial Park, Suzhou 215123, China

**Keywords:** micro electrochemical milling, machining localization, side gap, surface roughness

## Abstract

Micro electrochemical machining is becoming increasingly important in the microfabrication of metal parts. In this paper, the machining characteristics of micro electrochemical milling with nanosecond pulse were studied. Firstly, a mathematical model for the localization control of micro electrochemical milling with nanosecond pulse was established. Secondly, groups of experiments were conducted on nickel-based superalloy and the effects of parameters such as applied voltage, pulse on time, pulse period, electrolyte concentration and electrode diameter on machining localization and surface roughness were analyzed. Finally, by using the optimized machining parameters, some 2D complex shapes and 3D square cavity structures with good shape precision and good surface quality were successfully obtained. It was proved that the micro electrochemical milling with nanosecond pulse technique is an effective machining method to fabricate metal microstructures.

## 1. Introduction

With the development of Micro-Electro-Mechanical Systems (MEMS), demand for micro components and products is growing rapidly in fields such as electronics, optics, healthcare, automotive, biological, communications, and avionics industries. For example, MEMS sensors such as miniature navigation system, anti-maglev micro-gyroscope, and CMOS circuit microstructure are used in aerospace applications. MEMS integrated circuits such as amorphous silicon variable capacitor, micro resonators, and inverters are used in electronic communications and other fields. To meet this demand, a variety of microfabrication methods such as micro-cutting technology, lithography, micro electrical discharge machining (micro-EDM), micro electrochemical discharge machining (micro-ECDM) developed rapidly [1,2], alongside many notable achievements [3,4,5,6]. Micro-patterning can be performed with various techniques in MEMS. Electrochemical etching is actually a well-established technique developed initially for Si substrate (CMOS compatible). There is significant work performed for CMOS compatible processes for the creation of buried micro channels on Si employing electrochemical etching, micro fluidic flow sensors based on such micro channels. However, in the field of energy and aerospace, the common materials cannot withstand the harsh working conditions such as high temperature and pressure, large mechanical stress, violent sliding contact, corrosion, and high-intensity radiation. Therefore, these miniature components need to be made with high-performance metals and alloys, and they relate to micro grooves, micro cavities, and other complex three-dimensional structures. The nickel-based superalloy is generally considered as one of the most promising materials to solve this problem.

Nickel-based superalloy, which has good thermal stability, high strength, and hardness at high temperatures, anti-corrosion, and is durable among other characteristics, is a typical refractory material. It is widely used in the manufacture of the high-temperature components of aero-engine blades, rocket engines, nuclear reactors and energy conversion equipment. With the traditional cutting method for nickel-based superalloy, there will be some shortcomings such as tool making difficulties, cutting deformation, and generated cutting heat. Furthermore, the cutting force during the machining will cause the parts to deform, and the residual stress generated can also affect the quality and performance. Therefore, the traditional machining method is difficult to perform using the fine machining method required of these materials.

For the past few years, many experts and scholars have paid special attention to micro electrochemical machining technology, expecting to use its unique principles and characteristics to solve microfabrication problems. Micro electrochemical machining is an electrochemical dissolution process that has many advantages, such as no tool wear, stress-free, and smooth surfaces. It can also realize the machining with micron level precision compared with other techniques. Since the German MPG invented the nanosecond ultrashort pulse electrochemical machining in 2000 [7], the United States, Japan, Korea, and other industrialized countries in the world have invested heavily in micro electrochemical machining research. For example, Kunar machined a dimple array of micro holes with good geometrical shape [8]; Rathod used a disk-shaped electrode to conduct micro-electrolytic milling experiments, and successfully produced a series of microgrooves such as reverse tapered, barrel shaped, double stepped, and spherical [9]; Rathod researched the influence of electrochemical micro machining (EMM) parameters like tool feed rate, applied voltage, duty ratio, pulse frequency, and electrolyte concentration on the machining accuracy of a 500 μm-deep microgroove in stainless steel, and a high-quality microgroove with 55 μm width overcut and 10 μm-depth overcut with an aspect ratio of 2.31 was fabricated using an in situ-fabricated tungsten microtool of 110 μm diameter [10,11]; Yuan used current feedback to electrode position, and successfully fabricated array grooves with the width of 6 μm [12]; Xu added an adjustable inductance element in the equivalent circuit of the electrochemical micromachining to form a coupled fluid-electric circuit. With a tungsten electrode with a diameter of 15 μm, the multi-order 3D microstructure was obtained. The microgroove width was 16.8 μm, and the machining precision reached 900 nm [13]; Meng carried out a series of experiments on Ni-based metallic glass, optimized the experimental parameters, and produced a series of high quality metal glass microstructures, such as a micro curved cantilever beam, micro gear, and micro square helix [14,15]; Cole used signal-noise ratios and analysis of variance to optimize parameters of the electrochemical micromachining process, and the optimal holes with respect to the aspect ratio, surface roughness and the rate of electrochemical micromachining of Zr-based bulk metallic glasses (BMGs) were obtained [16]. Although the above research has its own achievements in the theory or experiment of EMM, there is still a lack of theoretical mathematical models and experimental research for a certain kind of micro ECM process, such as micro electrochemical drilling, milling or wire micro ECM. At present, the micro ECM technology, especially micro electrochemical milling is a long way from industrial application. The innovation of this paper is that the machining localization control model is established for the micro electrochemical milling process, which lays a foundation for follow-up experiments. Meanwhile, the in-depth analysis of the machining performance, including machining localization and machined surface quality, also provides a useful supplement for the complete evaluation of the electrochemical micro milling technology.

Good surface quality is critical for the final product and 3D structural production. In this article, a series of micro electrochemical milling experiments on a nickel-based superalloy were carried out. And the effects of parameters such as applied voltage, pulse on time, pulse period, electrolyte concentration and electrode diameter on machining localization and surface roughness were analyzed. Finally, the typical micro 2D and 3D structures were successfully machined by optimizing parameters, which has a good surface quality without a recast layer, heat affected zone (HAZ), and micro-cracking. This paper provides a basic technical support for the micro fabrication of the metal microstructure, which brings micro electrochemical milling technology closer to industrial application.

## 2. Principle of Micro Electrochemical Milling

**Figure 1:**
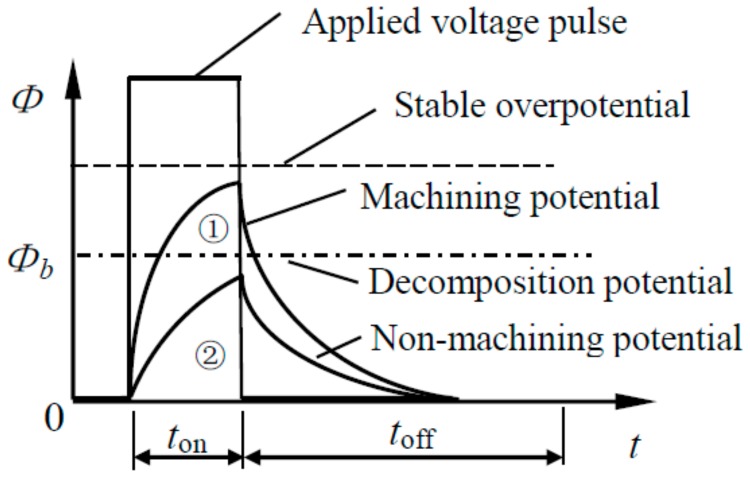
The double layer model.

The electrochemical reaction in electrochemical micromachining is driven by the potential difference of the electric double layer. When the ultrashort nanosecond pulse voltage is applied between the tool electrode and the workpiece material, the capacitance of the dielectric layer on the anode and cathode is periodically charged and discharged [17,18]. The area near the electrode will be charged strongly by the pulse voltage after the appropriate pulse width is selected. The overpotential φ will gradually increase, and the steady value will not be reached at the end of the pulse. This is a transient process. The area away from the electrode is significantly weaker. The electrochemical reaction speed varies exponentially along the electric potential drop on the double layer. A strong chemical reaction is only limited to a few microns in the region near the polarized electrode, so the machining localization of the nanosecond pulse electrochemical machining is high. Electrode potential changes are shown in Figure 1, where *Φ* is applied voltage, *t*_on_ is pulse on time, *t*_off_ is pulse interval, *Φ_b_* is decomposition potential of anode, *C_d_* is capacitance of the electric double layer, *R_f_* is resistance of electrochemical reaction, and *R_e_* is resistance of electrolyte.

**Figure 2:**
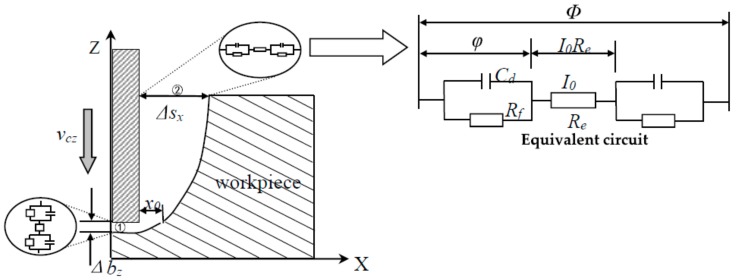
Sketch of feeding down.

**Figure 3:**
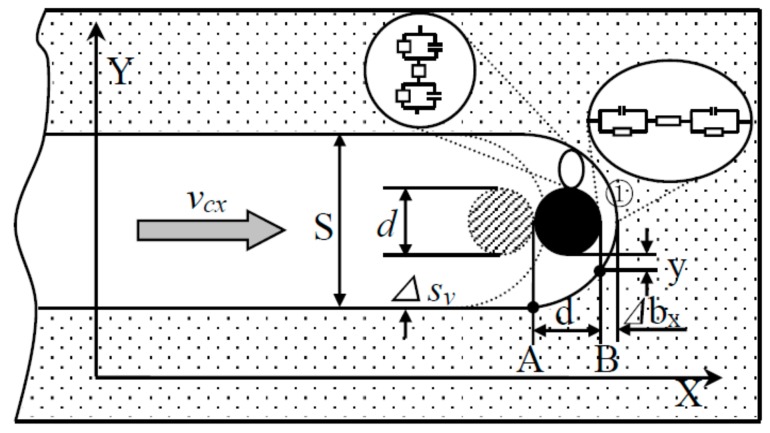
Sketch of feeding along XY axis.

The machining process of micro electrochemical milling by layer can be divided into two steps: (1) The electrode feed down along the Z-axis, as shown in Figure 2. (2) The electrode feed to plane mill along the X-Y plane trajectory after reaching the specified milling layer thickness, as shown in Figure 3. When the plane is finished, the electrode will go straight down. The workpiece is milled continuously by layer until the whole machining is finished.

(1) Feeding down

Figure 1 shows that the charging time of the double layer is very short during the process of nanosecond pulse electrochemical polarization. The change of overpotential φ of the electrode follows time t is as follows:

(1) $$\varphi =\varphi_{0}\left(1-\exp(-\frac{t}{\tau })\right)$$

where $\varphi_{0}$ is steady overpotential, τ is charging time constant is given by:

(2) $$\tau =R_{e}C_{d}=\frac{l}{\sigma }C_{d}$$

where the electrolytic resistance $R_{e}$ can be expressed as the product of the electrolyte resistivity 1/σ and the distance between electrodes l, $R_{e}=l/\sigma$.

When the machining process is balanced, the inter-electrode frontal gap $\Delta b_{z}$ is given by:

(3) $$\Delta b_{z}=\frac{\omega \sigma (\Phi -\delta E)}{v_{cz}}$$

where $v_{cz}$ is cathode feed speed. $v_{cz}$ is equal to the dissolution velocity at the bottom of the anode $v_{az}$ at that time.

When the cathode side is not insulated, the relational expression of the change of the side gap along the X-axis at the inlet of area ②:

(4) $$\frac{dx}{dt}=\frac{\omega \sigma (\Phi -\delta E)}{x}$$

That is:

(5) ∫xdx=∫ωσ(Φ−δE)dt

$x=x_{0}$ at t=0, solving the integral of Equation (5) gives:

(6) $$x=\sqrt{2\omega \sigma (\Phi -\delta E)t+x_{0}{}^{2}}$$

The overpotential of the electrode end and the face of the workpiece closed to the end wall are basically the same, so their electrochemical dissolution rate is roughly the same. Namely the initial gap $x_{0}\approx \Delta b_{z}$, then the inlet side gap $\Delta s_{x}$ can be given:

(7) $$\begin{array}{l} \Delta s_{x}=\sqrt{2\omega \sigma (\Phi -\delta E)t+\Delta b_{z}{}^{2}}\\ =\sqrt{\frac{2\omega \sigma (\Phi -\delta E)L_{z}}{v_{cz}}+{\left[\frac{\omega \sigma (\Phi -\delta E)}{v_{cz}}\right]}^{2}} \end{array}$$

where $L_{z}$ is electrode feed depth. The milling layer thickness $L=L_{z}+\Delta b_{z}$. That is:

(8) $$\Delta s_{x}=\sqrt{\frac{2\omega \sigma (\Phi -\delta E)L}{v_{cz}}-{\left[\frac{\omega \sigma (\Phi -\delta E)}{v_{cz}}\right]}^{2}}$$

The hole entrance diameter is determined by: $D=d+2\Delta s_{x}$, where d is the electrode diameter. Therefore, the hole entrance diameter is influenced by a variety of factors, such as the electrode diameter d, milling layer thickness L, feed rate $v_{c}$, applied voltage Φ, electrolyte conductivity σ, and so on.

(2) Feeding along the path

After the feed depth is determined, the electrode will feed along the Z-axis, then the X-Y plane milling is begun according to the predetermined trajectory, as shown in Figure 3. The derivation of the plane milling model can be calculated according to the derivation of step (1), so the side gap $\Delta s_{y}$ can be obtained as the following expression:

(9) $$\begin{array}{l} \Delta s_{y}=\sqrt{2d\Delta b_{x}+\Delta b_{x}{}^{2}}\\ =\sqrt{2d\frac{\omega \sigma (\Phi -\delta E)}{v_{cx}}+{\left[\frac{\omega \sigma (\Phi -\delta E)}{v_{cx}}\right]}^{2}} \end{array}$$

where $\Delta b_{x}$ is the inter-electrode frontal gap of area ① in Figure 3, $v_{cx}$ is the feed rate of the electrode along X-axis. So, the size of side gap depends on electrode diameter d, feed rate $v_{c}{}_{x}$, applied voltage Φ, electrolyte conductivity σ, and so on.

The groove width S is determined by:

(10) $$\begin{array}{l} S=d+2\Delta s_{y}\\ =d+2\sqrt{2d\frac{\omega \sigma (\Phi -\delta E)}{v_{cx}}+{\left[\frac{\omega \sigma (\Phi -\delta E)}{v_{cx}}\right]}^{2}} \end{array}$$

In order to ensure good shape and dimension precision, the machining precision parameters D in step (1) should have the following relationship with S in step (2): D≤S => $\Delta s_{x}\leq \Delta s_{y}$. That is:

(11) $$\frac{2\omega \sigma (\Phi -\delta E)L_{z}}{v_{cz}}+{\left[\frac{\omega \sigma (\Phi -\delta E)}{v_{cz}}\right]}^{2}\leq 2d\frac{\omega \sigma (\Phi -\delta E)}{v_{cx}}+{\left[\frac{\omega \sigma (\Phi -\delta E)}{v_{cx}}\right]}^{2}$$

To simplify the above model, the feed rate remains constant. That is when $v_{cz}=v_{cx}$, the expression above can be simplified as follows:

(12) $$L_{z}\leq d$$

In other words, when the feed rate is constant, the electrode feed depth $L_{c}$ along the Z-axis cannot be greater than the electrode diameter d to ensure better shape and dimensional precision.

## 3. Experimental System and Arrangement

**Figure 4:**
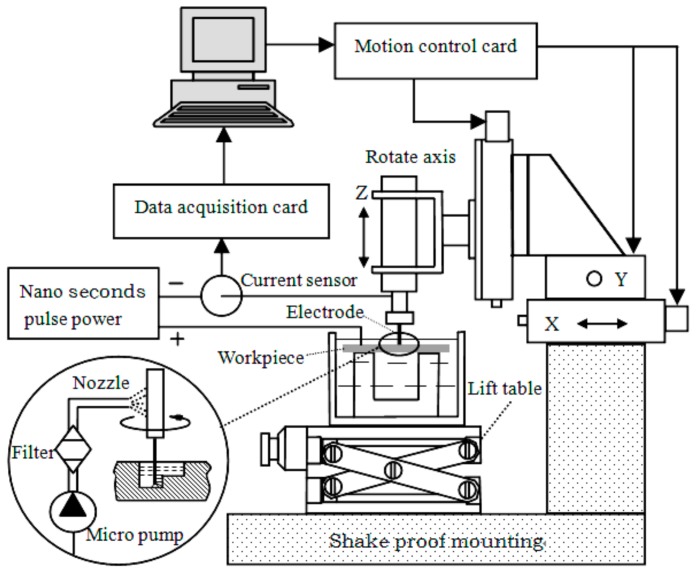
Schematic of the entire EMM setup.

This paper described a three-dimensional electrochemical micro milling experiment platform. The proposed EMM system, as shown in Figure 4, mainly includes a nanosecond pulse power unit, the electrode system, the motion control system, the machining state detection system, and the electrolyte circulating system.

The electrode system consists of a tool cathode, electrode clamps, high-speed spindle, and the workpiece. Micron grade cylindrical tungsten electrodes are selected as a tool cathode, which is connected by a screw thread between tool holder and the spindle. The high-speed spindle is clamped on the Z axis, and can move along with the X, Y, and Z three-axis movement. The workpiece is fixed in the electrolyte tank mounted on the lifting platform and can be moved up and down. In the experiments, the positive and negative pole of the nanosecond pulse generator was connected to the workpiece and tool electrode, respectively. A low concentration of acid solution was selected to flow in the circulatory system.

The motion control system consisted of a X, Y, Z three linear motion axis and the rotation axis C, which were driven by motor drives respectively. Rotation axis C is controlled by a VFD-B programmable controller (DELTA, Shanghai, China), speed continuous adjustable from 0 to 40,000 RPM, radial runout within 1 μm. The linear motion platform is taken from the Adlink company (New Taipei City, Taiwan) production of a MP-C154 four axis motion control card as the core, which can control the GMT (Taiwan) electric sliding table CYS-6020 (X, Y direction) and CXS-6030 (Z direction), realizing the three directions precision reciprocating feed of 0.1 μm/step resolution and a reciprocating positioning accuracy of ±0.5 μm, to meet the requirements of electrochemical micro machining.

The nanosecond pulse generator is selected as the power supply in the experiments, which can output the peak voltage of ±10 V, and the minimum pulse width of 8 ns. The detection system is mainly responsible for the current signal changes, which directly reflects the machining state. A small ohmic value resistor is a series connected in the circuit, and then the sensor detects the sampling resistance voltage value, which can be converted to the corresponding current value. Meanwhile, the multifunction data acquisition card NI PCI-6221 input signals are connected to the computer through the A/D converter. Through the analysis of the data processing in the computer, the real-time detection and feedback of the machining status is realized.

Stability is crucial for this type of micro machining with a gap of several micrometers. If the tool feed rate is too high, the tool will come in contact with the workpiece and cause a short circuit. Therefore, a safety system is necessarily built in the EMM set-up. When a short circuit is detected, the servo motor will stop and move backward along the path of the feed forward immediately until the danger is clear.

The appearance of the finished surface was examined with a three-dimensional profilometer (MicroXAM, ADE, Milpitas, CA, USA) and scanning electron microscope (NOVA NANOSEM 450, FEI, Hillsboro, OR, USA). The dimensions of the structures (length, width, and depth) were measured in the cross-sectional profile, which was obtained by a three-dimensional profilometer (MicroXAM, ADE, Milpitas, CA, USA).

Experiments of the micro electrochemical milling of a nickelbase superalloy (GH3030, Baosteel, Shanghai, China) plate with the thickness of 300 μm were carried out to demonstrate the effects of machining parameters on the side gap. As mentioned above, the side gap Δ*S* is considered as the evaluation of machining localization. The side gap is dependent on the electrode diameter, pulse voltage, pulse on-time, electrolyte concentration and so on. Table 1 shows the machining conditions for micro electrochemical milling.

## 4. Experimental Results and Analysis

### 4.1. Influence of Applied Voltage on Machining Localization and Surface Roughness

**Figure 5:**
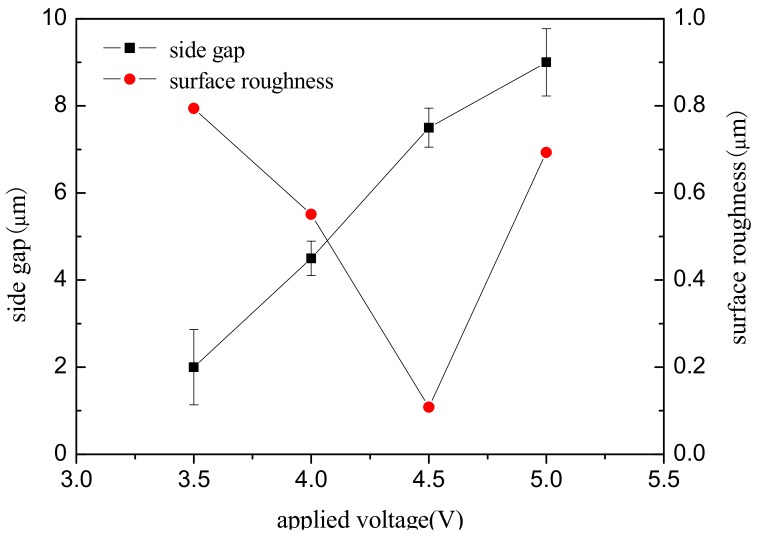
Effect of applied voltage on side gap and surface roughness.

To study the effect of voltage amplitude on the machining localization and surface roughness, a series of comparative experiments were conducted. The test parameters were as follows: 0.2 mol/L H_2_SO_4_ electrolyte, workpiece of a nickel-base superalloy (GH3030) plate with the thickness of 300 μm, pulse period of 1 µs, pulse on time of 95 ns, electrode diameter of 10 μm, feed rate of 0.2 μm/s, and depth of 10 μm. Figure 5 shows the variation of the side gap and surface roughness with an applied voltage on 3.5 V, 4 V, 4.5 V, and 5 V.

**Figure 6:**
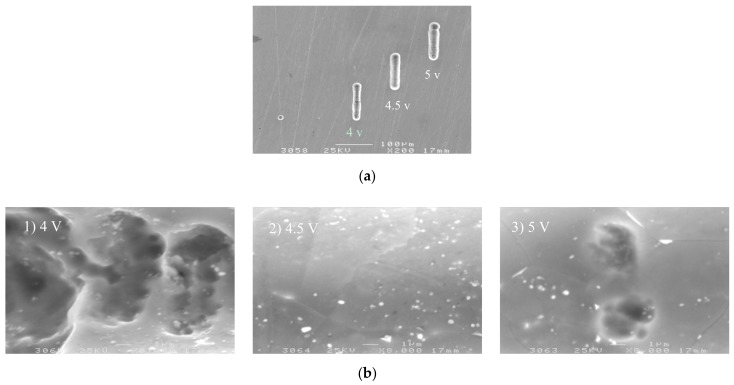
The microgroove under different voltages. (**a**) The SEM image of the microgroove; (**b**) The appearance of the microgroove.

It can be seen from Figure 5 that, as the applied voltage increases from 3.5 V to 5 V, the machining side gap increases, that is, the machining localization decreases with the increases in voltage. According to Equation (3), the side gap is proportional to the applied voltage when the workpiece material, electrolyte parameters, and feed rate remain constant. Therefore, the experimental results are consistent with the trend of theoretical change. The SEM image of the microgroove on different voltages is shown in Figure 6a. It can be seen that the voltage amplitude of 4 V can significantly improve the localization of the micro electrochemical milling than the 5 V.

The surface roughness of the bottom of the groove is obtained by three-dimensional profilometer (MicroXAM, ADE, Milpitas, CA, USA). Figure 6b is a comparison of the appearance topography of the microgroove under the voltage of 4 V, 4.5 V, and 5 V, respectively. The surface quality of the microgroove obtained by 4.5 V is the best. The surface quality is determined by two factors: machining energy in unit time and machining stability. On the one hand, the current density increases with the increase of machining voltage amplitude, and the surface quality reduces; On the other hand, excessive voltage amplitude reduces the machining stability. Therefore, to ensure better surface quality, it is necessary to reduce the voltage amplitude as far as possible under the premise of stable machining.

### 4.2. Influence of Pulse on Time on Machining Localization and Surface Roughness

To study the effect of pulse on time on machining localization and surface roughness, a series of comparative experiments were conducted. Applied voltage was 4.5 V, pulse on time ranged from 60 ns to 150 ns, and other parameters were the same as above.

**Figure 7:**
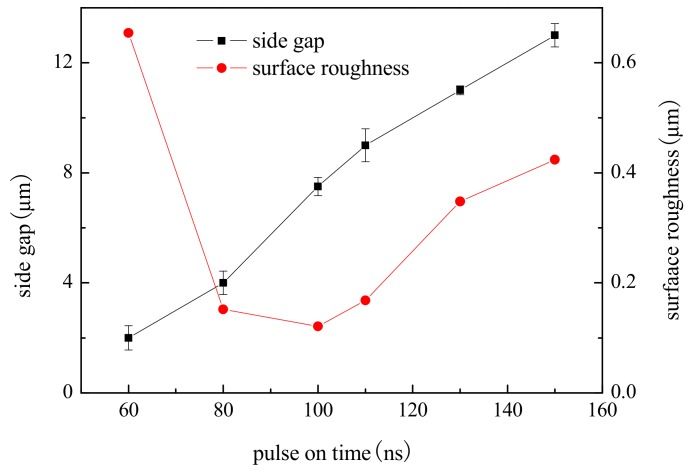
Effect of pulse on time on side gap and surface roughness.

**Figure 8:**
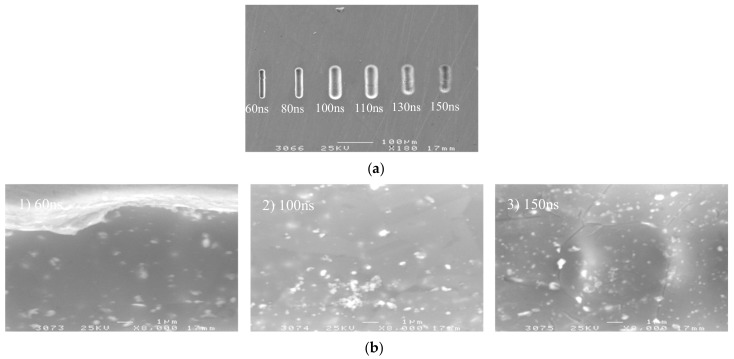
The microgroove under different pulse on times. (**a**) The SEM image of the microgroove; (**b**) The appearance of the microgroove.

Figure 7 shows the variation trend of the side gap and surface roughness with the pulse on time. The SEM image of the microgroove on different pulse on times is shown in Figure 8. It can be seen from the above illustration that, with the other parameters unchanged, the side gap increases with the increase of the pulse on time, and the machining localization decreases. In the experiment, when the pulse on time increased to more than 130 ns, the charging time of the double layer was prolonged, closer to the time constant of the non-machining part, which made the localization of the electrolytic machining drop. There was serious stray corrosion in this process, so the pulse on time should be reduced as far as possible. However, when the pulse on time is reduced to 60 ns, the electrode overvoltage is low, and the current density of the electrochemical reaction is small because the charging time of the double layer is too short. The dissolution rate of the workpiece material is too slow, which is less than the feed rate of the electrode, resulting in a significant decrease in the stability of the machining process. When the pulse on time is in the range of 80–100 ns, the machining process is stable and the microgroove has a high surface quality and sharp edges. Therefore, under the premise of guaranteeing the stability, generally 80–100 ns are taken.

### 4.3. Influence of Pulse Period on Machining Localization and Surface Roughness

To study the effect of the pulse period on machining localization and surface roughness, a series of comparative experiments were conducted. Pulse on time was 95 ns, pulse period ranged from 0.5 µs to 3 µs, and other parameters were the same as above.

**Figure 9:**
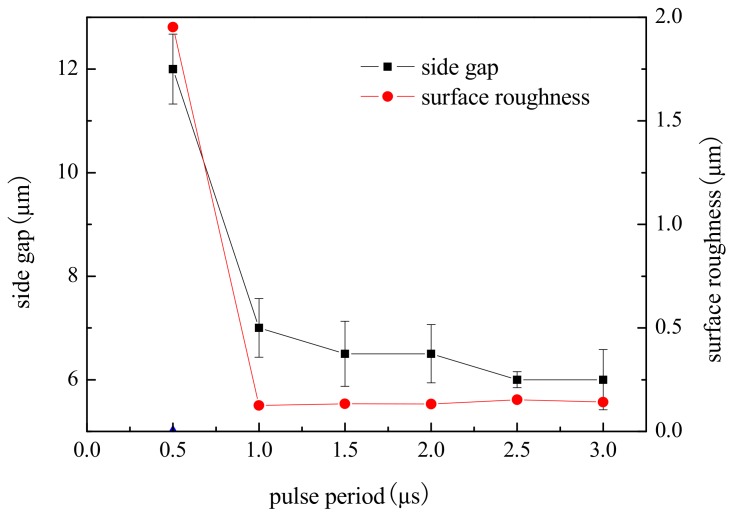
Effect of pulse period on side gap and surface roughness.

**Figure 10:**
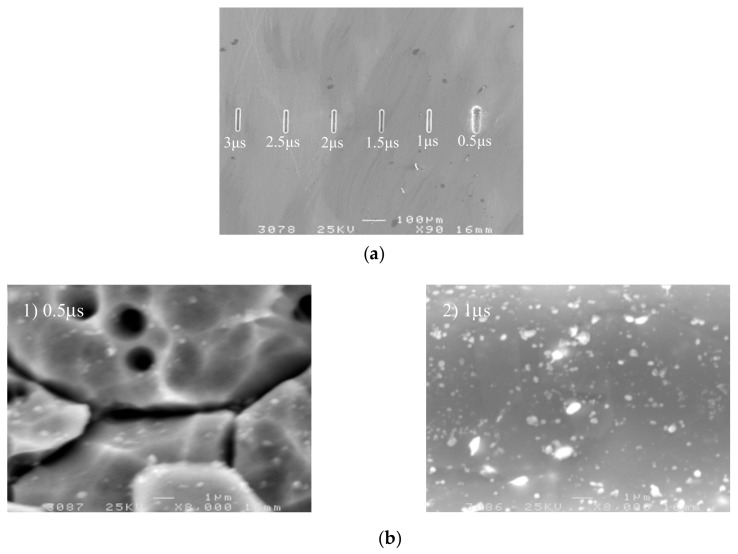
The microgroove under different pulse periods. (**a**) The SEM image of the microgroove; (**b**) The appearance of the microgroove.

Figure 9 shows the variation trend of the side gap and surface roughness with the pulse period. The SEM image of the microgroove on different pulse period is shown in Figure 10. As can be seen from the figure, the side gap generally decreases along with the increase of the pulse period. When the pulse period is 0.5 µs, the energy density is high per unit time, the material removal rate is high, and the side gap is large. However, the electrolytic products are not discharged in time, so the process is unstable, and the surface quality of the microgroove is poor. When the pulse period is more than 1.0 µs, the pulse interval is enough to ensure the electrolytic products are expelled under the condition of the same voltage and pulse on time. Thus, the side gap changes little and the machining stability is good. Consequently, in order to improve the efficiency and reduce unnecessary pulse interval time, we should try to take the smaller pulse period and generally take 1.0–3.0 µs.

### 4.4. Influence of Electrolyte Concentration on Machining Localization and Surface Roughness

To study the effect of electrolyte concentration on machining localization and surface roughness, a series of comparative experiments were conducted. Pulse period was 1 µs, electrolyte concentration ranged from 0.1 mol/L to 0.6 mol/L, and other parameters were the same as above.

**Figure 11:**
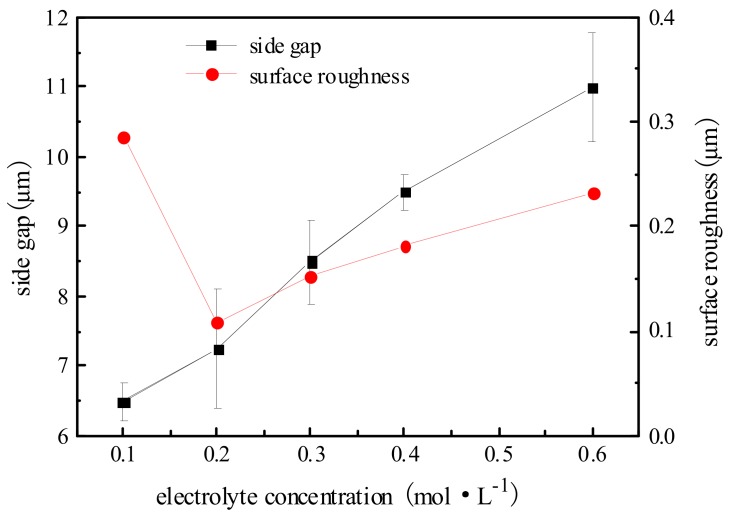
Effect of electrolyte concentration on side gap and surface roughness.

**Figure 12:**
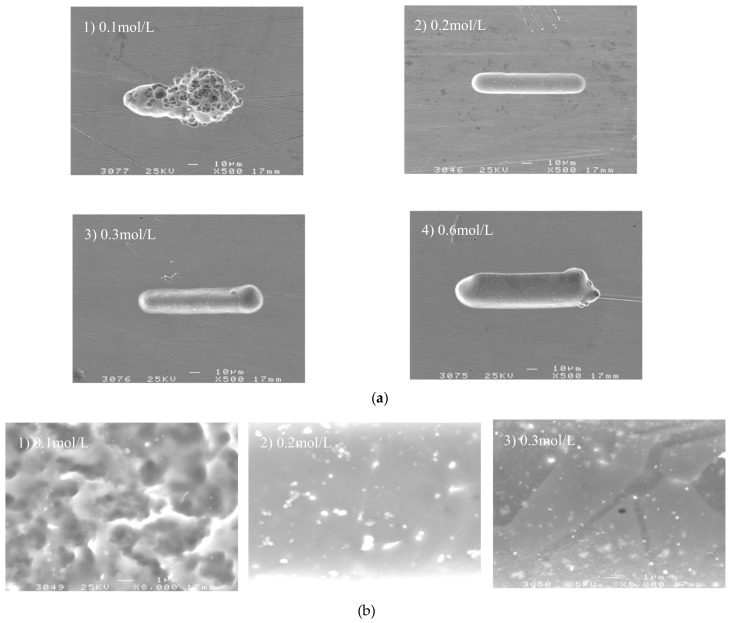
The microgroove under different electrolyte concentrations. (**a**) The SEM images of the microgroove; (**b**) The appearance of the microgroove.

Figure 11 shows the variation trend of the side gap and surface roughness with electrolyte concentration. The SEM image of the microgroove on different electrolyte concentrations is shown in Figure 12. It can be seen from the figure that, with the increase in electrolyte concentration, the side gap is significantly enlarged. This is because the change of electrolyte concentration will lead to the change of conductivity, thus the increase of conductivity will increase the side gap. And the increase of current density can make the surface quality worse, so the electrolyte at low concentration must be used as far as possible. However, when the electrolyte is at a low concentration of 0.1 mol/L, the electrolytic products cannot be dissolved in time due to the small concentration, attached to the electrode surface, which cause a sharp decrease in machining localization. Therefore, in order to obtain a good surface quality, the concentration of H_2_SO_4_ electrolyte is generally 0.2 mol/L under the premise of ensuring stable machining and certain machining efficiency.

### 4.5. Influence of Electrode Diameter on Machining Localization

To study the effect of electrode diameter on machining localization, a series of comparative experiments were conducted. Electrolyte concentration was 0.2 mol/L, electrode diameter ranged from 4 µm to 18 µm, and other parameters were the same as above.

**Figure 13:**
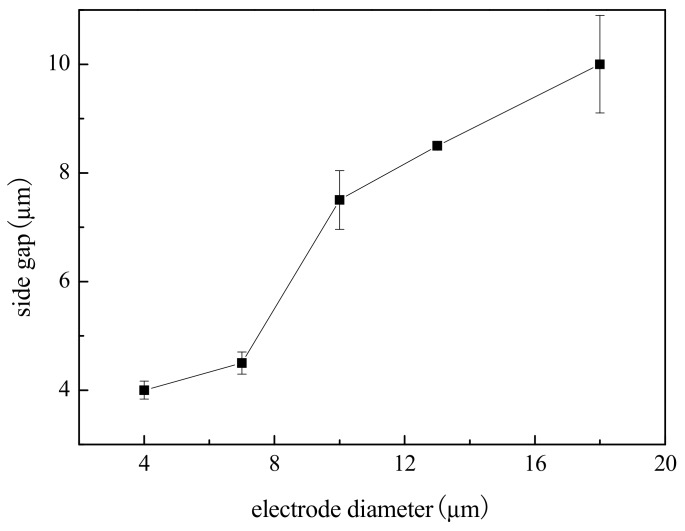
Effect of electrode diameter on side gap.

**Figure 14:**
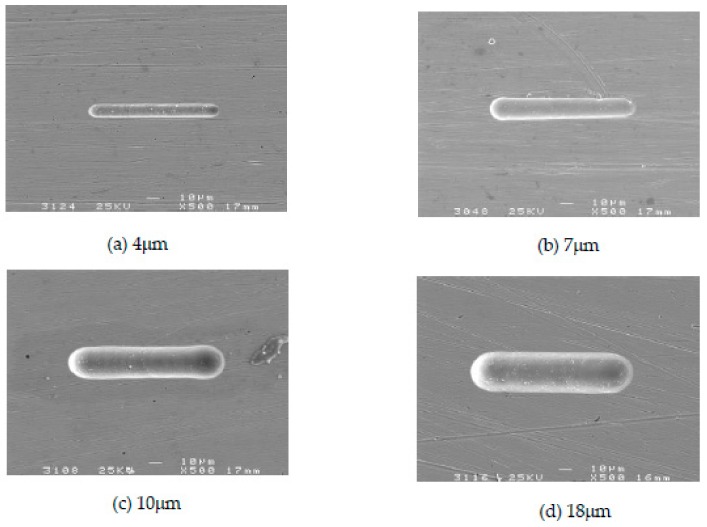
The SEM images of the microgroove under different electrode diameters. (**a**) 4 µm; (**b**) 7 µm; (**c**) 10 µm; (**d**) 18 µm.

Figure 13 shows the variation trend of the side gap with electrode diameter. The SEM image of the microgroove on different electrode diameters is shown in Figure 14. As can be seen from the figure that, the general trend is that the side gap increases rapidly with the increase of the electrode diameter, and the machining process is stable. Due to the constant machining parameters, there is no obvious change in the quality of the bottom surface after machining. In addition, the electrode diameter and the size of the side gap determine the width of the microgroove together. Therefore, the decrease of electrode diameter can reduce the width of the microgroove. In order to improve machining localization, choose an electrode with a small diameter for as far as possible.

### 4.6. Machining of 2D and 3D Complex Structures

**Figure 15:**
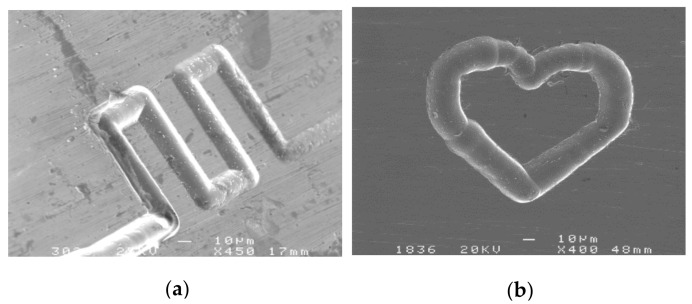
Two 2D complex micro-structures. (**a**) A micro-channel; (**b**) A heart-shaped structure.

Figure 15a shows a micro-channel with the width of 25 µm and the depth of 20 µm. The specific machining parameters were as follows: 0.2 mol/L H_2_SO_4_ electrolyte, workpiece of a nickel-base superalloy (GH3030) plate with the thickness of 300 μm, pulse period of 1 µs, pulse on time of 95 ns, electrode diameter of 10 μm, feed rate of 0.2 μm /s, and applied voltage of 4.5 V. Figure 15b shows a heart-shaped structure with the width of 25 µm and the depth of 20 µm, and the parameters are the same as Figure 15a.

It can be seen from the figure that micro electrochemical milling machining has the ability to machine micro and complex structures. The part of the curve is smooth, and the angular sharp is clear. The surface quality is good, and the bottom surface is smooth.

For the cavity machining with the complex structure and the high depth ratio, the machining depth is deep in micro electrochemical milling. The electrolyte cannot be updated in time, and the electrolyte products are difficult to discharge. Therefore, it is difficult to guarantee the machining localization and stability, and the efficiency is also very low. Thus, in order to obtain a good effect, the experiments adopt the machining by layer in which the complex structure with a high depth ratio is decomposed into a multi-layer simple structure. The thickness of each layer is controlled between 3 µm and 5 µm, so that the electrolyte can enter the machining gap in time and take away the electrolytic products. The short-circuit phenomenon caused by the accumulation of local products is eliminated and the stability is improved. In addition, the material removed in unit time is very little, and the machining at a higher speed can be used, which improves machining efficiency.

**Figure 16:**
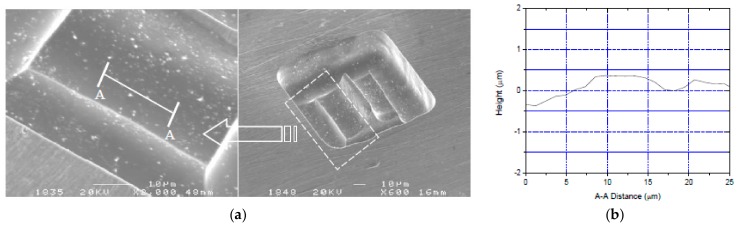
A 3D complex micro-structure. (**a**) A square cavity structure; (**b**) The cross section surface morphology.

A 3D square cavity structure with two-story steps was machined as shown in Figure 16a. The conditions are the same as above and the milling layer thickness was 3 μm. The total depth of the cavity was about 40 μm and the size of the tongue structure in the center was about 80 μm × 5 μm. After measurement and calculation, the finished surface roughness Ra of A-A cross-section as shown in Figure 16b is 0.125 μm. The outline and edges of the cavity are clear, and the milling plane is smooth uniformly. These features show high surface quality and good shape accuracy.

## 5. Conclusions

In this paper, a new method is introduced for fabricating micro metal parts with good shape precision and good surface quality, the conclusions can be summarized as follows:

- Based on the transient reaction process of the electrochemical double layer, the mathematical model of micro electrochemical milling was established, which lays a theoretical foundation for the subsequent experiments. That is: when the feed rate is constant, the electrode feed depth $L_{c}$ along the Z-axis cannot be greater than the electrode diameter d to ensure better shape and dimensional precision.
- The high precision micro-electrochemical machining platform was set up. Several experiments were carried out and the influence of applied voltage, pulse on time, pulse period, electrolyte concentration and electrode diameter on machining localization and surface roughness was analyzed. The side gap increases with the increase of applied voltage, pulse on time, electrolyte concentration, and the electrode diameter, and decreases with the increase of the pulse period. Then, the optimized parameters were obtained, which combined the effects of the surface roughness.
- Based on the optimization of the above parameters, the 2D complex shapes and the 3D square cavity structures were successfully machined, which have good shape precision and good surface quality. It is proved that the micro electrochemical milling with nanosecond pulse is an effective method that can meet the machining requirements of micro devices.

## Figures and Tables

**Table 1:** Machining conditions for EMM experiments.

Machining Conditions	Parameter
Applied Voltage (V)	3.5–5.5
Pulse on Time (ns)	60–150
Pulse Period (μs)	1
Electrode Diameter (μm)	3–20
Workpiece Material	Super alloy (GH3030)
Electrolyte	0.2M H_2_SO_4_ solution
Mill Layer Thickness (μm)	≤electrode diameter
Feed Rate (μm/s)	0.2–1
